# Estimation of Incidence and Prevalence of Pediatric Channelopathies in a Mediterranean Population Based on a Single-Center, Retrospective Analysis

**DOI:** 10.3390/children13050659

**Published:** 2026-05-08

**Authors:** Alena Bagkaki, John Papagiannis, Aris Anastasakis, Fragiskos Parthenakis, Gregory Chlouverakis, Emmanuil Galanakis, Ioannis Germanakis

**Affiliations:** 1 School of Medicine, University of Crete, 71 003 Heraklion, Greece; 2 Onassis Cardiac Surgery Center, Syggrou Av. 356, 176 74 Athens, Greece

**Keywords:** channelopathy, childhood, pediatric channelopathy, long QT syndrome, epidemiology, Crete, Mediterranean

## Abstract

**Highlights:**

**What are the main findings?**

- Long QT syndrome was the predominant channelopathy;
- Affected children represented the first diagnoses within families in half of the cases;
- Genetic testing was positive in 77% when performed;
- The epidemiology of pediatric channelopathies on the Mediterranean island of Crete seems similar to that reported in the literature, with regional clusters of increased prevalence.

**What are the implications of the main findings?**

- Screening children for channelopathies could potentially improve the detection of affected families;
- Knowledge of the epidemiology in selected populations can be helpful for the best design of large-scale screening programs in the pediatric population.

**Abstract:**

Background: Channelopathies represent a heterogeneous group of rare inherited cardiac diseases associated with life-threatening arrhythmias. Our knowledge of their epidemiology in childhood is limited. The aim of this study is to evaluate the epidemiology of pediatric channelopathies on a Mediterranean island (Crete, Greece). Methods: Retrospective study of children < 18 years followed in the Regional Tertiary Pediatric Cardiology Unit during a 24-year period (2002–2025) and meeting the disease-specific diagnostic criteria. Results: A total of 43 children (32 families) were enrolled, corresponding to an average annual incidence of 1.43 (95% C.I.: 1.03–1.92) and a cumulative prevalence of 31.1 (95% C.I.: 22.1–42.5) cases per 100, 000 children, with significant regional incidence differences. Long QT syndrome (n = 38) was predominant; rare cases of Brugada syndrome (n = 3) and Catecholaminergic polymorphic tachycardia (n = 2) were recorded. The diagnosis was based on symptomatic presentation (n = 15, 35%), while asymptomatic patients (n = 28, 65%) were diagnosed during cascade family screening (n = 22, 51%) and preparticipation screening (n = 6, 14%). They represented the first diagnosis within affected families (index cases) in 21/43 (49%) of cases. Genetic testing was performed in 35/43 (81%) channelopathy cases and it was positive in 33/43 (77%) of them, specifically in 30 out of 38 (79%) LQT cases with a genotype of LQT2 in 15 (39%), LQT1 in 10 (26%), LQT3 in one (3%) and LQT5 in two (5%) cases. Conclusions: The incidence of pediatric channelopathies on the Mediterranean island of Crete seems comparable to that reported in the literature, with regional clusters of significantly increased incidence. Further study of the epidemiology of pediatric channelopathies is needed, to document any regional or ethnic differences and for the best design of large-scale screening programs.

## 1. Introduction

Channelopathies are a heterogenous group of inherited, primary cardiac electrical diseases critically affecting the function of cardiomyocyte membrane ion channels, responsible for the action of potential generation (depolarization and repolarization) [1]. Although rare, with a reported prevalence ranging from 1/2000 to 1/5000 in the general population [2,3] with regional and ethnic variability [4], they are extremely important due to their associated significant morbidity and mortality, representing a main cause of sudden cardiac death (SCD) in young people [5,6,7]. Main clinical diagnoses include long QT syndrome (LQTS), Brugada syndrome (BrS), Catecholaminergic Polymorphic Ventricular Tachycardia (CPVT) and Short QT Syndrome (SQTS). Their early detection in childhood is of critical clinical importance as affected children might represent the first cases (index cases) in affected families [8], presenting with syncopal episodes resembling epilepsy, or even with sudden cardiac death [5,9,10]. Our knowledge of their epidemiology in childhood is rather limited, based mainly on a limited number of cross-sectional studies in different ethnic groups [2,11,12].

The Mediterranean island of Crete represents a part of Greece relatively isolated from the Greek mainland. It was inhabited since prehistory with a flourishing civilization interacting with other Mediterranean basin populations. Historical populations’ movements that occurred along the Mediterranean Sea had a crucial impact not only on the variability in prevalence, but also on the distinct genetic pattern of many hereditary diseases, compared to the Greek mainland [13]. The distinct epidemiology of pediatric cardiomyopathies on Crete has been recently reported [14]. The aim of the present study was to estimate, for first time, the population-based incidence and prevalence of pediatric channelopathies in a Mediterranean island population through a single-center, retrospective analysis.

## 2. Materials and Methods

This was a retrospective study of children younger than 18 years of age living in the region of Crete Island, evaluated in the Regional Academic Pediatric Cardiology Center during a 24-year period (January 2002 to December 2025), and meeting established disease-specific ECG/clinical diagnostic criteria for channelopathies [15,16]. The Pediatric Cardiology Unit, University Hospital Heraklion, represents the single academic referral center in Crete for pediatric patients, providing non-invasive diagnosis and treatment in close cooperation with the national tertiary referral center for pediatric electrophysiology (Onassis Cardiac Center—Athens); advanced electrophysiology services are offered by the Cardiology Department of our Institution for adults only. Data analyzed included cases diagnosed and followed up also in the regional referral center based on clinical indications or detected and referred through a pilot application of a pediatric cardiac screening program previously described [17]. The study was performed in line with the principles of the Declaration of Helsinki. The approval for collecting and processing data from the available medical records of each study patient was obtained from the University Hospital Heraklion, Ethics Committee, approval number 1007, 33/2 December 2020. Written informed consent for participation in research was obtained from parents/legal guardians of evaluated children < 18 years of age. All personal data were anonymized. None of Gen AI tools were used in any stage of data processing.

### 2.1. Study Population

The study population included children, who met disease-specific diagnostic criteria, according to 2022 and 2015 ESC Guidelines [15,16]. Disease-specific diagnostic criteria are summarized in Table 1.

### 2.2. Data Sources

Personal history, demographic characteristics, brief family history and related clinical symptoms—dizziness, palpitations, seizures, fainting episodes, syncope and aborted cardiac arrest—were collected from medical reports. Adverse outcomes including sudden cardiac death (SCD) were extensively searched and recorded if relative information was available on medical records. The diagnostic pathway included physical evaluation, standard blood chemical analysis, resting 12-lead electrocardiogram, 24 h Holter ECG monitoring, chest radiography and echocardiography in all cases. The duration of the QT interval was manually measured from the beginning of the QRS complex to the end of the T wave using the tangent method in lead II and V5, calculating the mean QT duration over three cycles [18].

The QTc interval was calculated with Bazett’s correction formula [19]. LQTS was suspected based on the three components of the Schwartz score—patient’s clinical history, family history, and ECG evaluation. Exercise testing on treadmill was performed in cooperative patients, evaluating the QTc duration at 4th minute of recovery period [20,21]. Positive results of already performed genetic evaluation were recorded.

Final diagnosis of long QT syndrome was established according to the clinical criteria endorsed in 2022 ESC Guidelines [15] confirmed by positive genetic testing in majority of them [22]. Under evaluation cases characterized by negative family history, borderline QTc prolongation, modified Schwartz score < 3 [16] and non-diagnostic (VUS or benign variants of disease-related genes) genetic testing [22,23,24,25] were excluded. Cases of transient QTc prolongation, associated with confirmed electrolyte abnormalities or use of QT prolonging drugs, were not included in this study. Asymptomatic phenotype-negative carriers of family-specific pathogenic variants of LQTS-, BrS- and CPVT-causing genes, detected during cascade family screening procedure, were included in this study according to diagnostic criteria (Table 1) [26,27,28]. Detailed family history and family pedigrees were documented. Clinical evaluation and cascade screening of 1st degree relatives was recommended.

### 2.3. Epidemiologic Data

The cumulative prevalence at the last year of study enrollment and the average annual incidence rate of channelopathies in the pediatric population of Crete were estimated, including documented cases meeting the disease-specific diagnostic criteria. Regional differences in incidence of channelopathies were evaluated as well. All demographic data were obtained from the Hellenic Statistical Authority Office [29].

### 2.4. Statistical Analysis

The incidence (new diagnoses per 100,000 children < 18 years of age per year, for each year of the duration of the study) and the prevalence (total cases per 100,000 children < 18 years) were estimated by the retrospective data analysis. The Hellenic Statistical Service data, regarding total and regional (total of 4 Prefectures) pediatric population of the island of Crete, were used as denominators to calculate the total and regional annual incidence and prevalence of channelopathies [29]. Data were summarized as frequencies and percentages for categorical data and as medians and means for age of presentation. Confidence intervals (95% C.I.) were estimated using the online software MedCalc version 23.5.3 [30]. Regional differences regarding demographic and clinical variables were evaluated with appropriate methods for qualitative data (Chi-Square Test, Fisher exact test, Odds Ratio, Relative Risk and 95% C.I.) and numerical data (t-Test, Mann–Whitney U test paired comparisons, ANOVA or Kruskal–Wallis test group comparisons, for variables with normal or non-normal distribution, respectively). SPSS program (IBM SPSS Statistics V26) was used for data analysis. Significant differences (*p* < 0.05) were documented.

Regional differences in incidence of channelopathies were statistically analyzed using MedCalc online software [30] for comparison of two rates as follows: incidence in each region [number of LQTS cases in a region as nominator/total person–years (children population < 18 Y over 24 years) in corresponding region as denominator] was compared separately to relevant regional incidence in other regions; significant differences (*p* < 0.05) were documented. None of Gen AI tools were used in any stage of data statistical analysis.

## 3. Results

**Figure 1:**
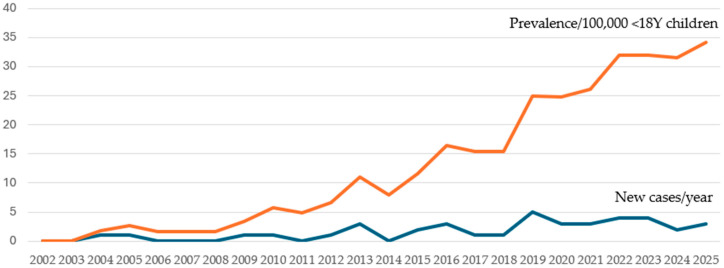
Pediatric channelopathies, Crete, Greece. New cases per year (2002–2025) and annual prevalence/100,000 children.

Over a 24-year period (2002–2025) 43 new cases of pediatric channelopathy were documented. The average island population during this period was 615,000, including 125,300 children under 18 years of age. The average annual incidence of pediatric channelopathies in Crete was estimated at 1.43 (95% C.I.: 1.03–1.92) patients per 100,000 children, although with significant geographical differences. The cumulative prevalence in the last year of enrollment was estimated at 31.1 (95% C.I.: 22.1–42.5) patients per 100,000 children < 18 years old. The predominant channelopathy was long QT syndrome, with a prevalence of 30.3 (95% C.I.: 21.4–41.6) cases per 100,000 children. Table 2 presents the average annual incidence and prevalence at last year of enrollment of channelopathies, while Figure 1 presents the annual incidence and cumulative prevalence, as documented for each year of the study period. Estimated prevalence of pediatric long QT syndrome reported in epidemiological studies based on mass screening programs of neonates and/or school-aged children are presented in Table 3.

Diagnoses: From the total of 43 diagnoses, 38 cases corresponded to long QT syndrome (LQTS), three cases to Brugada syndrome and two cases to Catecholaminergic Polymorphic Ventricular Tachycardia (CPVT). Sex: There were 20 (47%) boys and 23 (53%) girls. The difference in sex distribution was not statistically significant (*p* = 0.5). Age: Mean (median) age of diagnosis was 7.3 (7.6) years (range 0.1–15). Mean (median) age of diagnosis in boys was 7.1 (six) years, in girls 7.5 (eight) years. This difference was not statistically significant (*p* = 0.4).

Geographical distribution: A statistically increased annual incidence of channelopathies, reaching 2.46 cases per 100,000 children per year, was documented in the Middle-Western Region, compared to other island’s regions, as presented in Table 4.

Diagnostic approach: The diagnosis was based on symptomatic presentation (n = 15, 35%), preparticipation screening (n = 6, 14%) or family cascade screening (n = 22, 51%). Patients were diagnosed as index cases in 49% (n = 21) of cases. Symptoms at diagnosis included dizziness n = 5 (11%), syncopal episodes n= 8 (19%) and seizures n = 2 (5%), while 28 cases (65%) were asymptomatic.

Family history: The total of 43 channelopathy cases belong to 32 families; however, due to the relationship between some of them (n = 5) an increased number of affected members were concentrated in the same geographical region. A positive diagnosis in at least one first-degree relative was recorded in 29/43 (67%) cases. The probability of an affected parent was reported in 16/43 (37%) cases (mother in n = 8 cases, father in n = 8 cases), and the probability of an affected sibling was reported in 7/43 (16%). Positive history of sudden cardiac death (SCD) was recorded in 8/43 (18%) cases, and in all instances, this occurred in second-degree relatives.

Genetic evaluation: Genetic testing was performed in 35/43 (81%) channelopathy cases and it was positive in 33/43 (77%) of them.

Cascade family screening: A total of 62 children (corresponding to 28 families) were evaluated to rule out the presence of pediatric channelopathy in the context of cascade family screening following confirmed diagnosis in family members. A total of 22 children (corresponding to 18 families) were diagnosed following cascade screening, corresponding to 35% of evaluated children (and 64% of corresponding families). Genetic testing was performed in 36/62 (58%) of children undergoing cascade screening, which was positive in 21/62 (34%) of cases.

Type of Channelopathy:

(A) Long QT syndrome. LQTS was the most frequent pediatric channelopathy, documented in 38 cases. The average annual incidence was estimated at 1.26 (95% C.I.: 0.9–1.7) per 100,000 children per year, while the cumulative prevalence in 2025 was estimated at 30.3 (95% C.I.: 21.4–41.6) cases per 100,000 children. Sex distribution was as follows: 15 (40%) boys and 23 (60%) girls. The mean age at diagnosis was 7.1 years (range 0.1–15). Genetic testing was performed in 30/38 (79%) of LQTS patients. Positive genotype was confirmed in n = 28/38 (74%) LQT cases. Pathogenic variants in the following LQT-related genes—KCNQ1, KCNH2, SCN5A and KCNE1—were found and consequently the following four LQT genotypes were determined: LQT1 in n = 10/38 (26%), LQT2 in n = 15/38 (39%), LQT3 in n = 1/38 (3%) and LQT5 in n = 2/38 (5%) cases. Diagnosis was established in the first year of life in n = 5 (13%) cases and in n = 2 (5%) cases; it was associated with a severe phenotype (recorded symptomatic Torsade de Pointes episodes or recurrent syncopal episodes).

(B) Brugada syndrome. No symptomatic patient was documented; three asymptomatic genotype positive children were included, who were detected during cascade family screening with known family-specific pathogenic variants of BrS genes (SCN5A and KCNJ8 genes) detected in their symptomatic parents. The mean age at the initial evaluation was 8 years (range 5–10), while the mean duration of follow-up was 6 years. The spontaneous Brugada type 1 pattern was absent in all ECG tracings during follow-up of all evaluated children.

(C) CPVT. Two cases were documented. One symptomatic case was diagnosed after an episode of symptomatic bidirectional ventricular tachycardia (RYR2 gene). One asymptomatic case involving a pathogenic variant of RYR2 gene was detected during genetic screening for familiar cardiomyopathy [14].

(D) No case of SQT syndrome was documented.

Therapy: Standard recommendations regarding avoidance of QT prolongation agents and sport participation according to current guidelines were recommended [35,36]. Medical treatment of LQT patients was genotype-specific (when genotype was available), including beta blockers, and sodium channel blocker (Mexiletine) in LQT3 patients [37]. Complex therapeutic management in n = 2 (5%) patients (LQTS and CPVT) included antiarrhythmic treatment and surgical therapeutic methods (left-sided or bilateral sympathetic denervation) followed by ICD implantation. Asymptomatic BrS children did not receive any treatment. Symptomatic CPVT patient received additional beta blocker therapy [15].

## 4. Discussion

The Mediterranean region is characterized by an increased prevalence of many hereditary diseases [13]. Our study group has documented an increased prevalence of pediatric cardiomyopathies on the Mediterranean island of Crete [14]. To our knowledge this is the first study reporting the epidemiology of pediatric channelopathies in the Southeastern Mediterranean region. The annual average incidence of channelopathies in the pediatric population on Crete is estimated at 1.43 cases per 100,000/year. The prevalence of channelopathies and LQTS is estimated at 31.3 cases and 30.3 cases per 100,000 children (<18 years) respectively.

Long QT syndrome is the most frequent channelopathy [3,38,39] and the only one with a well-reported prevalence due to available large-scale epidemiological studies in neonates and infants from Italy [2,31] and Japan [32], and in children from Japan [11,34]. LQTS prevalence in neonatal period is estimated between 38/100,000 [2] and 42/100,000 [31] in Italy, and at 23.3/100,000 [32] in Japan, which corresponds to one case per 2000–2500 children. We emphasize that the age group of our cohort as well as the methods used to arrive at our estimates are quite different from the estimates derived purely from neonate- and infant-based screenings.

The results from large-scale, systematic screening programs among school-aged children in Japan are more comparable to the results of the present study. The prevalence of LQTS in the 6–12-year age group was reported by Yoshinaga et al. [11] to be 30–93 cases per 100,000 children, while in the study by Hayashi et al. [34], the prevalence of LQTS in the 6-to-12-year age group was 37.6 cases per 100,000 children. In the present study, the estimated prevalence of LQTS was 30.3 cases per 100,000, corresponding to one case per 3300 children. Care should be taken when comparing these figures, as the data of the present study are not based solely on the results of mass pediatric population screening programs. In our study only a limited number of primary school children (944) were screened and one single diagnosis of LQT syndrome was established [17].

A slight predominance of girls (60%) in this study correlates with the results of the Japanese [11] and Dutch studies [40]. The distribution of channelopathies by age correlates with the distribution of that reported in the literature for pediatric LQTS cases. In this study 13% of cases were diagnosed during the first year of life and were associated with severe clinical symptoms, in accordance with published data [41,42]. The diagnosis of LQTS in infancy and preschool age was approximately equally distributed. The age of elementary school students is considered by some authors to be the most suitable age for implementing screening programs, based on 12-lead ECG recording [11,34], in contrast to the early detection of LQTS in infancy, preferred by other authors [2,43,44].

In the present study regional differences in incidence of LQTS cases were documented. An increased incidence, detected in the Middle-West area of the island of Crete, could be a random phenomenon, but the presence of genetically distinct populations due to the residents of these areas being relatively isolated from the wider population of the island is also possible. Given that there may be carriers of family-specific “private” gene mutations in channelopathies, this should be considered for further investigation [45,46] in future studies.

Pediatric LQTS cases presented in this study were either symptomatic at diagnosis or LQTS diagnosis was established in asymptomatic children during cascade family screening or pre-participating screening procedure. Nearly two thirds of LQTS patients were asymptomatic at diagnosis. Targeted family screening and sports pre-participation screening contributed to the diagnosis of channelopathies in our population similar to previous studies [47,48]. Screening of all family members, regardless of the presence of symptoms, as well as extending of screening to second- or third-degree relatives, significantly increases the effectiveness of screening procedure. In our study, cascade family screening, including clinical, echocardiography and targeted genetic testing, became an effective method for identifying nearly half of affected children. High yield of family screening in the early diagnosis of LQTS was confirmed by a nationwide Danish study, where 228 affected relatives (children and adults) from 90 families were identified [47].

Although not all of our cases underwent genetic testing evaluation, DNA analysis has become an integral part of every patient diagnostic evaluation. In our study, genetic testing was performed in 81% of children and positive results were confirmed in 77% of them. Genetic testing in channelopathies is reported to be positive in 75–80% of LQTS cases [24,49], in 66% of CPVT cases [26], and only in 25–30% cases of Brugada syndrome [27,28]. The diagnostic yield of genetic testing in LQTS in this study (positive diagnoses among cases who underwent genetic testing) was close to reported data from other studies [8]. In our study LQT2 represented the most frequent genotype (39%). In Rochester LQTS registry, LQT1 predominated (46% vs. 26% in the present study), LQT2 followed (42% vs. 39%) with LQT3 being the least common form (12% vs. 3%) [50]. In the Italian LQTS registry LQT1 also predominated (56%) followed by LQT2 (32%) and LQT3 (12%) [51]. The predominance of LQT2 in the pediatric population of Crete might represent a geographic variation in pediatric LQT epidemiology [4].

In our study two cases of CPVT were found. CPVT is a rare, highly malignant channelopathy. The rarity of CPVT is confirmed by a limited number of recorded cases in international databases [26,52]. Patients with CPVT often present with exercise or emotion-induced ventricular tachycardia leading to syncope or cardiac arrest. The heart is structurally normal and the ECG in rest has no pathognomonic feature.

Brugada syndrome is a rare disease in young patients compared with adult populations and the prevalence in children and the young remains not well defined [27]. Symptoms associated with Brugada syndrome include fainting, syncope or seizures that often happen during febrile conditions [27]. In our study we did not document any symptomatic pediatric BrS case, but systematic follow-up of genotype-positive children regarding experience of specialized centers is provided [53,54].

Reported pediatric cases of Short QT Syndrome are extremely rare [55], similar to our study where no patient was detected.

The present study findings should be viewed within their limitations. Our study is based on retrospective analysis of available medical records information, compared to the majority of available studies in this field, which represent cross-sectional studies corresponding to large-scale ECG screening programs. Prevalence estimates based on neonatal and/or school-based screenings (summarized on Table 3) might not be directly comparable to the prevalence reported in this study. The present study, a retrospective study of 24 years of medical records, includes a combination of symptomatic cases, cascade family screening and children identified from a preliminary application of pilot primary school screening program. Prevalence estimates based on neonate and infant screening could be different from the age-spectrum of the present study, which includes all children aged < 18 years. Therefore, the possibility of individual cases failing to be registered cannot be ruled out while further undetected cases among the reference population cannot be excluded.

Our academic Pediatric Cardiology Unit represents the single established facility of pediatric cardiology services on the island, offering non-invasive diagnosis and treatment from fetal life up to adolescence to our island’s pediatric population. There are a few private pediatric cardiology praxis on the island, mainly involved in preparticipation screening for children, while basic services can be offered as a first-line evaluation by adult cardiologists to four national health system regional hospitals. Given that the Regional Academic Center for Pediatric Cardiology is the only referral center for pediatric patients with cardiovascular diseases on the island of Crete, it was unlikely that symptomatic infants or children suspected of channelopathy would not be referred to this center for evaluation and follow-up monitoring. Despite excellent cooperation with all above facilities, which refer their cases to our institution, the possibility that limited families might seek directly advice and care elsewhere (enrolment bias) cannot be ruled out. In our study the probability of relative underestimation of asymptomatic LQTS pediatric patients belonging to yet undetected familiar clusters cannot be ruled out.

The term “incidence”, in the context of inherited disorders (when applied to cascade family screening) in this and previous studies, might be better expressed as “annualized diagnostic incidence” as biological incidence (truly captured at birth) is different from “diagnostic” incidence.

Genetic testing is an important part of the diagnostic procedure in all familiar cardiovascular diseases. However, the availability of genetic testing is subject to financial restrictions, while the interpretation of positive results as VUS or as mutations of unknown significance requires updated information and appropriate consultation [23]. In our study the majority of patients with positive diagnostic criteria revealed pathogenic or likely pathogenic mutations when genetic testing was performed.

## 5. Conclusions

Based on this single-center, retrospective analysis, the incidence of pediatric channelopathies on the Mediterranean island of Crete seemed comparable to that reported in published literature. Further population-wide neonatal, infant or school-based screenings are needed to provide more accurate estimates of prevalence and incidence of pediatric channelopathies as well as to detect any regional or ethnic differences.

## Figures and Tables

**Table 1:** Channelopathies—disease-specific diagnostic criteria [15,16].

	Diagnostic Criteria
Long QT Syndrome	(a) **QTc ≥ 480 ms** by Bazett’s formula in repeated 12-lead ECG with or without symptoms in the absence of a secondary cause of LQTS. (b) **QTc > 460** in repeated 12-lead ECGs in a patient with unexplained syncope or aborted cardiac arrest in the absence of a secondary cause for QT prolongation. (c) **LQTS risk score** (“Schwartz score”) > 3. (d) Presence of **pathogenic mutations in LQTS genes** irrespective of QTc duration.
Brugada Syndrome	(a) **ECG: Spontaneous or induced** (fever, provocative testing) **type 1 (“coved”)** pattern is considered the only diagnostic pattern for BrS regardless of clinical symptoms. (b) Presence of **pathogenic mutation in BrS-causing genes**.
Catecholaminergic Polymorphic Ventricular Tachycardia (CPVT)	(a) The presence of normal resting ECG in a structurally normal heart and **exercise- or emotion-induced bidirectional VT or PVT (polymorphic ventricular tachycardia)**. (b) Presence of **pathogenic mutation in CPVT-causing genes**.
Short QT Syndrome	(a) **QTc ≤ 360 ms** in the presence of a pathogenic mutation and/or a positive family history of SQTS in the absence of secondary causes of SQTS. (b) SQTS should be considered in the presence of a **QTc ≤ 320 ms**.

**Table 2:** Epidemiology of pediatric channelopathies 2002–2025, Crete, Greece.

	Cases (N)	Incidence/ 100,000 Children < 18 Y/Year (95% C.I.)	Prevalence/ 100,000 Children < 18 Y (95% C.I.)
Total	43	1.43 (1.03–1.92)	31.1 (22.1–42.5)
LQTS	38	1.26 (0.9–1.7)	30.3 (21.4–41.6)
BrS	3	0.09 (0.02–0.3)	2.4 (0.5–6.9)
CPVT	2	0.06 (0.01–0.2)	0.8 (0.02–4.4)

Abbreviations. LQTS: long QT syndrome; BrS: Brugada syndrome; CPVT: Catecholaminergic Polymorphic Ventricular Tachycardia.

**Table 3:** Epidemiological studies of pediatric long QT syndrome.

Long QT Syndrome	Schwartz [2] Circulation 2009	Nosetti [31] Clinics and Practice 2024	Yoshinaga [32] Circ Arrhythm Electroph 2013	Simma [33] Neonatology 2020	Hayashi [34] Clinical Science 2009	Yoshinaga [11] European Heart Journal 2016	
Retrospective/prospective	Prospective	Retrospective	Prospective	Prospective	Prospective	Prospective	
Duration of study	2001–2006	2001–2017	2010–2011	2015–2018	2004–2005	2008–2013	
Methodology	Neonatal ECG screening	Neonatal ECG screening	Neonatal ECG screening	Neonatal ECG screening	Population-based ECG screening	Population-based ECG screening	
Country	Italy	Italy	Japan	Germany Regensburg	Japan Kanazawa	Japan Kagoshima	
Age of children	15–25 days	20–40 days	4 weeks	27 days	6–12 years	6 years	12 years
Number of children screened	44,596 infants	42,200 neonates/young infants	4285 young infants	2251 neonates	7961 school-aged children	32,982 1st-grade students	34,572 7th-grade students
LQTS cases (positive diagnostic criteria)	17	27	1	2	3	10	32
Prevalence (cases per 100,000)	38	42	23.3	88	37.6	30	93
Sex distribution (boys/girls) (%)	51/49	40/60	60/40	51/49	51/49	50/50	47/53

**Table 4:** Epidemiology of pediatric channelopathies in Crete, Greece. Regional annual incidence.

	Crete Total Cases/ Average Incidence (95% C.I.)		Western Region Cases/ Average Incidence (95% C.I.)		Middle-West Cases/ Average Incidence (95% C.I.)		Central Region Cases/ Average Incidence (95% C.I.)		Eastern Region Cases/ Average Incidence (95% C.I.)	
Total	N = 43	1.43 (1.03–1.92)	N = 6	0.81 (0.3–1.7)	N = 11	2.46 * (1.2–4.4) *p* = 0.02	N = 25	1.66 (1.1–2.4)	N = 1	0.3 (0.008–1.8)
LQTS	N = 38	1.26 (0.9–1.7)	N = 6	0.81 (0.3–1.7)	N = 10	2.24 * (1.1–4.1) *p* = 0.04	N = 21	1.4 (0.9–2.1)	N = 1	0.3 (0.008–1.8)
BrS	N = 3	0.09 (0.02–0.3)	N = 0		N = 0		N = 3	0.2 (0.04–0.6)	N = 0	
CPVT	N = 2	0.07 (0.01–0.2)	N = 0		N = 1	0.22 (0.005–1.2)	N = 1	0.06 (0.001–0.3)	N = 0	

Abbreviations: LQTS: long QT syndrome; BrS: Brugada syndrome; CPVT: Catecholaminergic Polymorphic Ventricular Tachycardia; * *statistically significant difference compared with all other regions*.

## Data Availability

The data presented in this study are available from the corresponding author upon request. The data are not publicly available due to privacy and ethical restrictions.
